# *En face* optical coherence tomography detection of Schlemm’s canal in primary open angle glaucoma

**DOI:** 10.3389/fphys.2023.1214427

**Published:** 2023-11-02

**Authors:** Haili Huang, Lijia Tian, Xinghuai Sun, Yuhong Chen

**Affiliations:** ^1^ Department of Ophthalmology and Visual Science, Eye and ENT Hospital, Shanghai Medical College, Fudan University, Shanghai, China; ^2^ NHC Key Laboratory of Myopia, Shanghai Key Laboratory of Visual Impairment and Restoration, Chinese Academy of Medical Sciences, Fudan University, Shanghai, China; ^3^ State Key Laboratory of Medical Neurobiology and MOE Frontiers Center for Brain Science, Institutes of Brain Science, Fudan University, Shanghai, China

**Keywords:** Schlemm’s canal, primary open angle glaucoma, swept source optical coherence tomography, three-dimension, *en face*

## Abstract

**Purpose:** To compare the morphological characteristics of Schlemm’s canal (SC) in patients with primary open-angle glaucoma (POAG) and healthy controls, using swept-source optical coherence tomography (SS-OCT) with *en face* reconstruction.

**Methods:** In this Prospective comparative study, we included 100 eyes from 50 patients diagnosed with POAG and 50 healthy controls. Three-dimensional cube and line scans of the temporal and nasal quadrants of the anterior segment of the limbus were acquired using SS-OCT. SC was identified using *en face* and cross-sectional images. The diameter and area of SC in cross-sectional images and the visible percentage and area of SC in *en face* images were measured using ImageJ.

**Results:** SC was observed in 84% of *en face* images and 81% of cross-sectional images in eyes with POAG but in 92% of *en face* images and 86% of cross-sectional images in control eyes. Significant differences between the POAG and normal control eyes were found in the *en face* area (0.35 ± 0.14 mm^2^ vs. 0.56 ± 0.22 mm^2^ in the temporal quadrant and 0.36 ± 0.14 mm^2^ vs. 0.58 ± 0.23 mm^2^ in the nasal quadrant; both *p* < 0.001) and visible percentage of SC (85.71% vs. 94.91% and 87.10% vs. 95.52% in the temporal and nasal quadrant respectively, both *p* < 0.001) in *en face* images as well as the cross-sectional area (2790.9 ± 942.2 μm^2^ vs. 4138.6 ± 2027.8 μm^2^ in the temporal quadrant and 2805.7 ± 947.2 μm^2^ vs. 4224.0 ± 2002.2 μm^2^ in the nasal quadrant, both *p* < 0.001) and diameter of SC (123.1 ± 25.4 μm vs. 149.5 ± 34.7 μm in the temporal quadrant and 126.3 ± 28.9 μm vs. 155.3 ± 36.0 μm in the nasal quadrant, both *p* < 0.001) in cross-section images. In addition, the mean intraocular pressure (IOP) significantly correlated with the *en face* area, visible percentage of SC, and cross-sectional area in the temporal and nasal quadrants.

**Conclusion:** SS-OCT can obtain high-quality *en face* images of SC without post-acquisition processing. Eyes with POAG had a decreased *en face* SC area compared with normal eyes. A correlation between SC area, visible percentage of *en face* images, and IOP was also observed.

## 1 Introduction

Glaucoma is the leading cause of irreversible blindness worldwide (Flaxman et al., 2017). Primary open-angle glaucoma (POAG), a major subtype of glaucoma, is highly prevalent and affects over 60 million adults globally (Zhang et al., 2021). The foremost risk factor for POAG is elevated intraocular pressure (IOP), which is affected by aqueous humor outflow. The elevated outflow resistance of the aqueous humor leads to an elevated IOP. The primary pathway for aqueous humor outflow is via the trabecular meshwork, followed by Schlemm’s canal (SC), collector channels, and scleral veins, which is responsible for the most of the aqueous humor drainage (Dautriche et al., 2015; Lewczuk et al., 2022).

SC is a circular channel in the eye that lies directly adjacent to the juxtacanalicular trabecular meshwork in the outer portion of the inner scleral sulcus. It is an elongated ellipse with its longer axis measuring 150–350 μm on cross-section (Lewczuk et al., 2022). Accumulating studies (Johnstone and Grant, 1973; Van Buskirk, 1982; Johnson and Kamm, 1983; Allingham et al., 1996; Kagemann et al., 2014a) have reported constriction or progressive collapse of SC in glaucomatous eyes, which may be responsible for increased aqueous outflow resistance and elevated IOP. Furthermore, surgical procedures have been developed to target mechanical dilation of SC or removal of the inner walls of SC to lower IOP (Okamoto et al., 2022). Therefore, SC structure imaging is helpful in facilitating therapeutic decision-making and evaluating the efficacy of glaucoma surgeries in patients with POAG.

Many different imaging techniques, such as confocal and light microscopy (Battista et al., 2008), high-frequency ultrasound biomicroscopy (Yan et al., 2016), micro-computed tomography (Hann et al., 2011), and anterior segment optical coherence tomography (AS-OCT) (Hong et al., 2013), have been used to evaluated the structure of SC. AS-OCT is a non-contact imaging technique that enables high-resolution three-dimensional visualization and real-time assessment of SC (Ang et al., 2018). Several studies (Chen et al., 2013; Li M et al., 2017; Chen et al., 2018b; Chen et al., 2018a) have reported *in vivo* SC imaging using OCT, and most of them used OCT B-scan imaging to measure the cross-sectional dimensions of SC in the nasal and/or temporal quadrants. On B-scan OCT, SC appears as a thin, black lucent space located in the deeper area of the corneoscleral limbus. Studies (Wang et al., 2012; Hong et al., 2013; Kagemann et al., 2014b) have indicated that POAG patients have smaller cross-sectional SC dimensions. Conventional B-scan OCT can only image a small segment of SC at a time, which may not reflect the overall condition of SC. *En face* OCT, on the other hand, can image a larger area of SC in one view, providing a more comprehensive assessment of SC structure. However, the analysis of SC using *en face* OCT imaging has not been previously reported.

Accordingly, we aimed to evaluate the clinical use of *en face* OCT in characterizing *in vivo* SC microstructures and to investigate inter-individual and regional variations between POAG and normal eyes.

## 2 Materials and methods

This prospective cross-sectional observational study was approved by the Ethics Committee of Eye and ENT Hospital, Fudan University, Shanghai, China. All study methods adhered to the tenets of the Declaration of Helsinki. Written informed consent was obtained from all participants.

### 2.1 Participants

Fifty patients diagnosed with POAG between August 2021 and June 2022 at the Department of Ophthalmology, Eye and ENT Hospital of Fudan University were included. To avoid the effect of ametropia on SC measurement, patients with POAG and normal participants whose myopic refractive error <−3D or > 3D were excluded. The diagnostic criteria for POAG included IOP >21 mmHg, characteristic glaucomatous visual field abnormality compatible with a retinal nerve fiber layer defect, and a peripheral anterior chamber depth deeper than one-fourth of the corneal thickness. Patients with systemic diseases and those who underwent prior ocular surgeries or had a history of ocular trauma or other eye diseases, including secondary glaucoma, were excluded from the study. Age-matched healthy volunteers without ocular or systemic conditions that could affect SC structures or compromise the OCT image quality were recruited. For all participants, a detailed medical history was obtained, and ophthalmic examinations were performed on the same day before the enrollment and imaging, which included visual acuity, refractive error, slit-lamp examination, Goldmann applanation tonometry, fundus photography, central corneal thickness, and axial length measurement using a LENSTAR Optical Biometer (Haag-Streit, United States). In addition, visual field testing (Humphrey perimetry) was also done. Each test was performed by a single examiner.

### 2.2 Measurements

**FIGURE 1:**
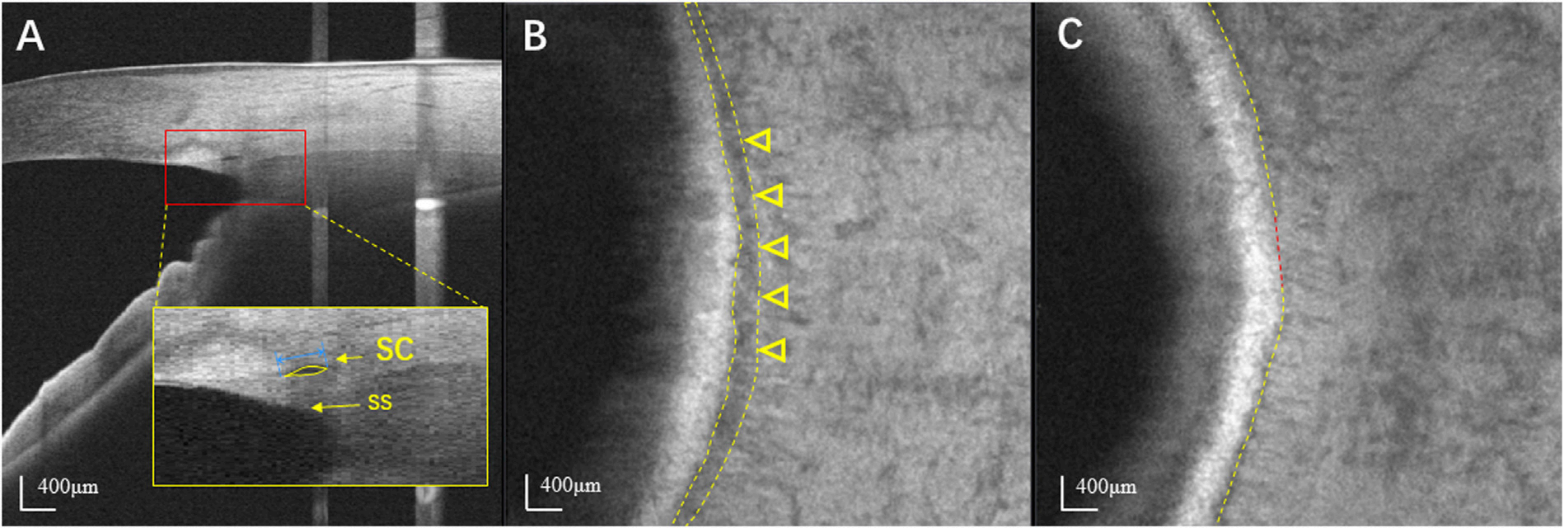
Identification of SC using swept-source optical coherence tomography. **(A)** The horizontal B scan image of SC in a normal eye. SC is seen as black, lucent ellipsoid space outside of the trabecular meshwork. The SS appears wedge shaped, resembling a spur. The SC diameter was measured from the anterior to the posterior endpoint of SC (blue line). The SC area was drawn freehand and was depicted as the area surrounded by the outline of SC (yellow arrow). **(B)** *En face* image at the level of SC in the same normal eye. SC is seen as a dark curved line parallel to the corneal limbus (yellow arrowheads). **(C)** The visible percentage of SC was defined as the percentage of the length of the visible SC (yellow line) to its total length. SC: Schlemm’s canal; SS: Scleral spur.

The AS-OCT instrument used in this study was a high-resolution swept-source optical coherence tomography (SS-OCT) system (VG200D, SVision Imaging, Henan, China) with a wavelength of 1,050 nm, scan speed of 100,000 A-scans per second, imaging depth of 6 mm in the tissue, lateral resolution of 10 µm and axial resolution of 5 µm. Participants were seated and instructed to stare at a nasal or temporal fixation light to ensure that the iridocorneal angle was centered in the instrument’s field of view. The scans were performed independently at the 3 and 9 o’clock positions with the B-scan model of a 6 mm line and at the nasal and temporal quadrants with a 6 × 6 mm^2^ anterior segment cube model containing 256 × 256 axial scans. A built-in automatic evaluation system software was applied to assess the quality of images, considering factors such as signal strength, contrast, and noise. Each site was scanned three times, and the image with the best quality at each position was selected for the final analysis. *En face* OCT images were created by built-in automated software (van Gogh v2.1.016; SVision Imaging, Henan, China). The software aligns and averages the cross-sectional images from cube model scans. This visualization displays the volume data on the XY plane while projecting it in the axial (Z) direction. Scleral spur was chosen as a reference point to identify the layer of SC. The depth and thickness of *en face* projection were manually adjusted by observers. The projection calculation was performed within that specific range for optimal visualization of SC. Digital images were processed by a single operator using the ImageJ software (ImageJ 1.53q, National Institutes of Health, Bethesda, MD). The SC diameter was measured from the posterior to the anterior SC endpoints in the B-scan cross-sections. The SC area was drawn freehand and depicted as the area surrounded by its outline (Figure 1A). In *en face* images, the *en face* area (EFA) of SC was also drawn freehand based on its outlines (Figure 1B). The visible percentage of SC was calculated as the percentage of the length of the visible SC to the total length of SC (Figure 1C). For each image, SC parameters in each quadrant were assessed and quantified manually by two independent and well-trained observers.

### 2.3 Data analysis

When both eyes met the diagnostic criteria, one eye was randomly selected for analysis, and qualified OCT images were acquired. All continuous variables are presented as mean ± standard deviation and categorical variables are as counts and percentages for each category. Inter-group differences were determined using the independent sample *t*-test (continuous data) or Fisher’s exact test (categorical data). The clinical data and morphological features of SC were compared between eyes with POAG and normal control eyes. Pearson’s correlation coefficient was used to determine the association between SC parameters and IOP. The intraclass correlation coefficient (ICC), which measures the proportion of total variability in measurements contributed by the variability in measurements between different participants, was determined using the random-effects mixed model. All statistical analyses were performed using the SPSS software (SPSS Statistics version 22.0; IBM Corp., Armonk, NY, United States). Statistical significance was defined as *p* < 0.05.

## 3 Results

### 3.1 Demographic characteristics of patients

One hundred individuals, including 50 POAG patients (50 eyes, 50%) and 50 healthy controls (50 eyes, 50%), participated in this study. The POAG group included 27 (54%) males and 23 (46%) females, whereas the control group included 26 (52%) males and 24 (48%) females. The mean age (mean ± SD) of the POAG and control groups was 42.74 ± 9.16 years and 40.16 ± 11.13 years, respectively. The mean IOP at imaging was significantly higher in the POAG group (20.21 ± 3.82 mmHg) than that in the control group (14.84 ± 2.62 mmHg). The mean refractions were −1.38 ± 1.19 D and −1.19 ± 1.18 D, the central corneal thickness (CCTs) were 541.18 ± 30.79 µm and 544.94 ± 25.57 µm, and the axial lengths were 23.64 ± 0.68 mm and 23.49 ± 0.66 mm in the POAG and control groups, respectively. The two groups showed no significant differences in sex, mean age, refractive error, CCT, or axial length. These data are summarized in Table 1.

**TABLE 1:** Clinical characteristics of study subjects.

Parameters	POAG	Normal controls	*p*
Number of patients (eyes)	50 (50)	50 (50)	-
Mean age (SD), years	42.74 (9.16)	40.16 (11.13)	0.209
Gender, male/female	27/23	26/24	1.000^a^
Number of anti-glaucoma drops			
0	11	50	
1	39	0	
IOP (SD), mmHg	20.21 (3.82)	14.84 (2.62)	<0.001*
Refraction (SD), D	−1.38 (1.19)	−1.19 (1.18)	0.425
CCT (SD), μm	541.18 (30.79)	544.94 (25.57)	0.508
Axial length (SD), mm	23.64 (0.68)	23.49 (0.66)	0.237
MD, dB	−8.13 (5.41)	−0.22 (0.14)	<0.001*

POAG: primary open-angle glaucoma; IOP: intraocular pressure; D: diopters; CCT: central corneal thickness.

^a^ Fisher’s exact test.*p < 0.05 indicates statistical significance.

### 3.2 Comparison of SC parameters between POAG and normal controls

The number and proportion of eyes in which SC was observable are summarized in Table 2. SC was a black lucent ellipsoid space outside the trabecular meshwork on cross-section images and a dark curved line parallel to the corneal limbus in *en face* images, which was defined as observable based on both examiners’ judgments. *En face* images were reconstructed using a commercially available automated algorithm based on SS-OCT. The percentages of *en face* images with detectable SCs were 84% and 92% in the POAG and control groups, respectively, without a statistically significant difference between the temporal and nasal quadrants (*p* = 0.357). In cross-sectional images, the differences in the percentage of detectable SCs between the nasal and temporal quadrants were not significant in either POAG patients or normal controls. The intraclass correlation coefficient of SC parameters measured by the same observer (HH) was 0.95, and that measured by different observers (HH and TL) was 0.90. The repeatability and reproducibility of these parameters were excellent.

**TABLE 2:** Proportion of eyes with detectable SC in the POAG and the normal control groups.

	POAG	Normal controls	*p*
Enface image			
Temporal quadrant, *n* (%)	42/50 (84)	46/50 (92)	0.357
Nasal quadrant, *n* (%)	42/50 (84)	46/50 (92)	0.357
*p*	1.000	1.000	
Cross-sectional image			
Temporal quadrant, *n* (%)	40/50 (80)	42/50 (84)	0.795
Nasal quadrant, *n* (%)	41/50 (82)	44/50 (88)	0.577
*p*	1.000	0.774	

POAG: primary open-angle glaucoma; EFA: *en face* area; CSA: cross sectional area.

*p* < 0.05 indicates statistical significance.

**FIGURE 2:**
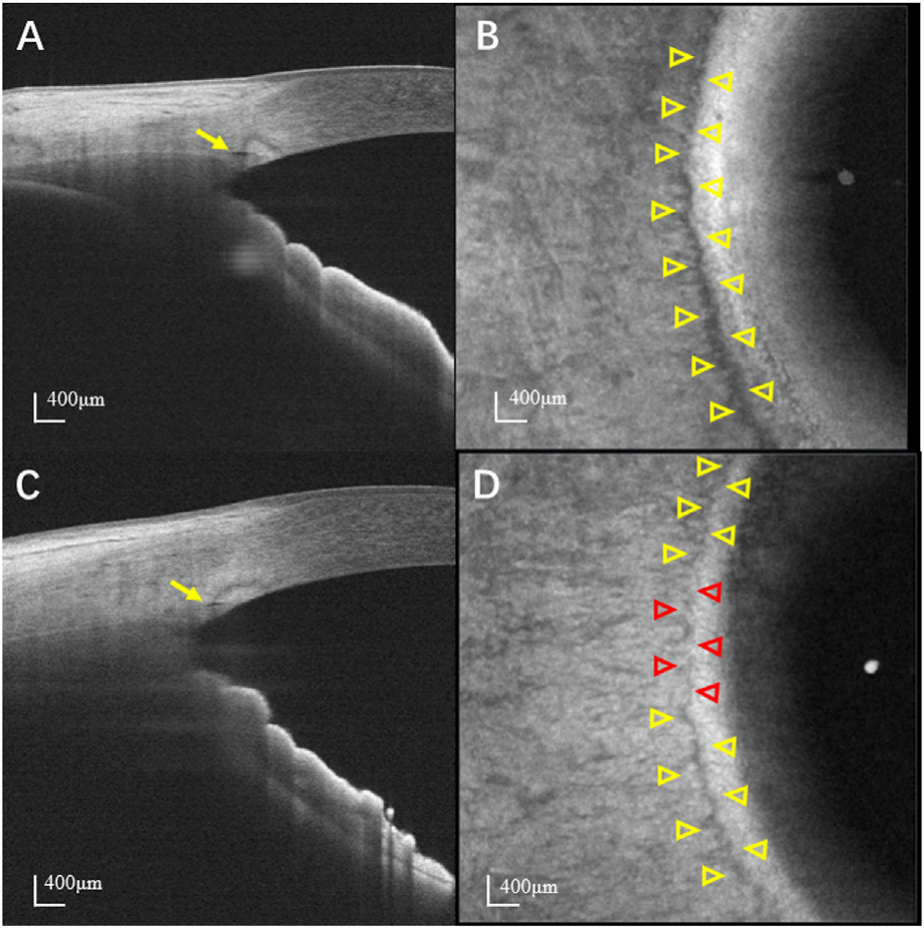
Representative images of SC. **(A)** Cross-sectional and **(B)** *en face* image of a normal eye. **(C)** Cross-sectional and **(D)** *en face* image of a POAG eye. Yellow and red arrowheads indicate the detectable and non-detectable SC, respectively. The SC is more prominent and continuous in normal eyes than that in POAG eyes. SC: Schlemm’s canal; POAG: Primary open angle glaucoma.

Significant differences in SC parameters were observed between patients with POAG and normal controls (Table 3). Based on the *en face* images, the areas of SC in the temporal and nasal quadrants were significantly smaller in POAG patients (0.35 ± 0.14 mm^2^ in temporal quadrant and 0.36 ± 0.14 mm^2^ in nasal quadrant) than in normal controls (0.56 ± 0.22 mm^2^ in temporal quadrant and 0.58 ± 0.23 mm^2^ in nasal quadrant; both *p* < 0.001). The visible percentages of SC in the temporal and nasal quadrants of POAG patients (85.71% ± 14.26% in temporal quadrant and 87.10% ± 14.39% in nasal quadrant) were significantly lower than those of normal controls (94.91% ± 7.45% in temporal quadrant and 95.52% ± 6.70% in nasal quadrant; both *p* < 0.001). Based on cross-sectional images, the diameters of SC in the temporal and nasal quadrants in patients with POAG were also smaller than those in normal controls. In detail, the areas of SC in cross-sectional images of SCs in the POAG and control groups were respectively 2790.9 ± 942.2 µm^2^ and 4138.6 ± 2027.8 µm^2^ in the temporal quadrants and 2805.7 ± 947.2 µm^2^ and 4224.0 ± 2002.2 µm^2^ in the nasal quadrants (both *p* < 0.001). The diameters of SC in the temporal and nasal quadrants were respectively 123.1 ± 25.4 µm and 126.3 ± 28.9 µm in POAG patients and 149.5 ± 34.7 µm and 155.3 ± 36.0 µm in normal controls (both *p* < 0.001). Representative SC images obtained from control and POAG eyes are shown in Figure 2.

**TABLE 3:** The parameters of SC in the POAG and normal control groups.

Parameters	POAG	Normal controls	*p*
Temporal quadrant			
EFA, mm^2^	0.35 (0.14)	0.56 (0.22)	<0.001*
Visible percentage, %	85.71 (14.26)	94.91 (7.45)	<0.001*
CSA, µm^2^	2790.9 (942.2)	4138.6 (2027.8)	<0.001*
Diameters, µm	123.1 (25.4)	149.5 (34.7)	<0.001*
Nasal quadrant			
EFA, mm^2^	0.36 (0.14)	0.58 (0.23)	<0.001*
Visible percentage, %	87.10 (14.39)	95.52 (6.70)	<0.001*
CSA, µm^2^	2805.7 (947.2)	4224.0 (2002.2)	<0.001*
Diameters, µm	126.3 (28.9)	155.3 (36.0)	<0.001*

*p* < 0.05 indicates statistical significance. EFA: *en face* area; CSA: cross sectional area.

### 3.3 Correlation between IOP, MD and SC parameters

**FIGURE 3:**
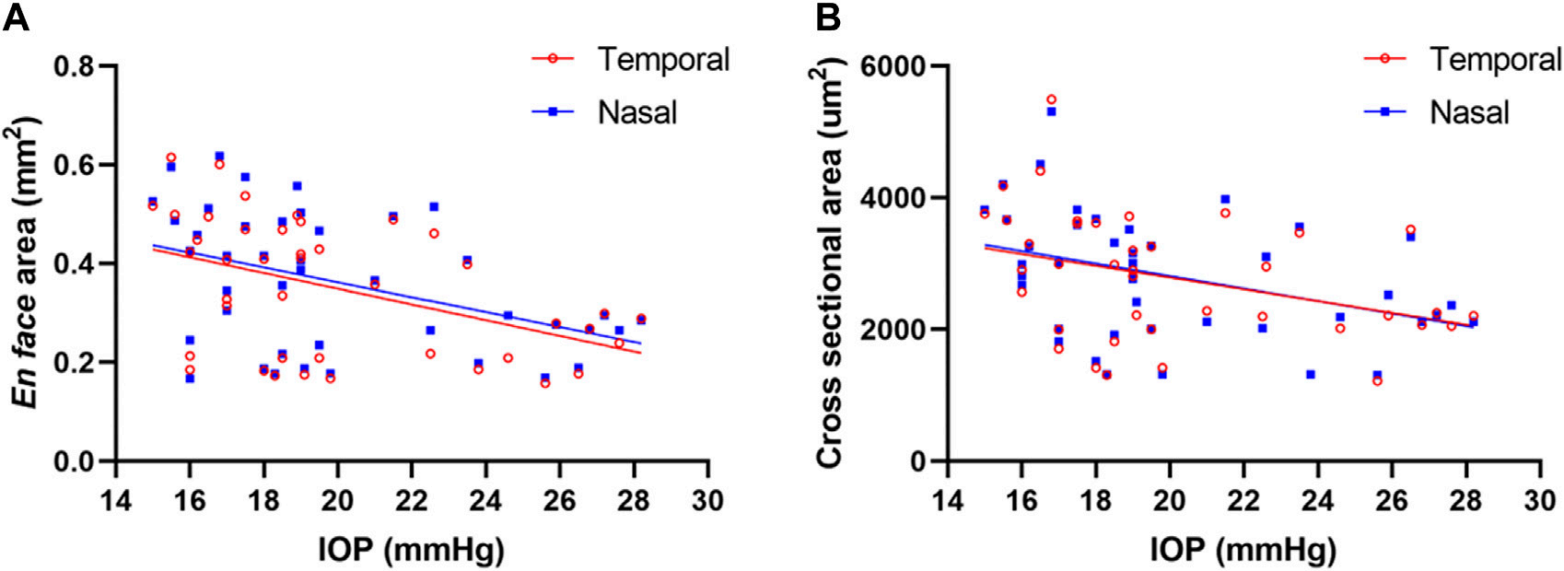
Correlations between EFA, CSA, and IOP. The EFA **(A)** and CSA **(B)** in the temporal and nasal quadrants in all POAG subjects were negatively correlated with IOP in the POAG patients with statistical significance. EFA: *en face* area; CSA: cross-sectional area; IOP: intraocular pressure.

The associations of IOP, mean deviation (MD) of the visual field, and SC parameters in the POAG group are presented in Table 4 and Figure 3. There was a negative correlation between IOP and *en face* area (*r* = −0.453 and *p* = 0.003 in temporal quadrant, *r* = −0.426 and *p* = 0.005 in nasal quadrant) and visible percentage of SC (*r* = −0.357 and *p* = 0.020 in temporal quadrant, *r* = −0.357 and *p* = 0.020 in nasal quadrant). Meanwhile, IOP was also statistically significantly correlated with the cross-sectional area of SC in temporal quadrant (*r* = −0.368, *p* = 0.019) and nasal quadrant (*r* = −0.390, *p* = 0.012) in cross-section images. However, the diameters of SC in cross-sectional images had no correlation with IOP in either the temporal quadrant (*r* = −0.161, p = 0.322) or the nasal quadrant (*r* = −0.216, p = 0.175). Furthermore, no significant correlations were found between the MD of visual field and SC parameters (*p* > 0.05).

**TABLE 4:** Correlations between IOP, MD and SC parameters.

Parameters	IOP		MD	
	r	*p*	R	*p*
Temporal quadrant				
EFA	−0.453	0.003*	−0.054	0.732
Visible percentage	−0.357	0.020*	−0.117	0.462
CSA	−0.368	0.019*	−0.114	0.483
Diameters	−0.161	0.322	−0.075	0.646
Nasal quadrant				
EFA	−0.426	0.005*	−0.041	0.796
Visible percentage	−0.357	0.020*	−0.173	0.274
CSA	−0.390	0.012*	−0.094	0.560
Diameters	−0.216	0.175	−0.082	0.609

*p < 0.05 indicates statistical significance. EFA: *en face* area; CSA: cross sectional area.

## 4 Discussion

*En face* imaging showed that eyes with POAG had smaller SC diameters and areas and decreased visible percentages of SC compared with normal eyes. In addition, correlation analysis showed a significant relationship between IOP and SC parameters in both cross-sectional and *en face* images. These findings demonstrate the value of *en face* imaging using SS-OCT for identifying SC structures. To the best of our knowledge, this study is the first to describe and characterize the morphological features of SC in eyes with POAG using *en face* OCT.

SC is a circular channel in the eye that drains the majority of aqueous humor from the anterior chamber into the bloodstream (Dautriche et al., 2015; Lewczuk et al., 2022). SC’s shape, size, and function depend on the intraocular pressure (IOP). High IOP can cause SC to collapse (Battista et al., 2008; Grieshaber et al., 2010), the trabecular meshwork to herniate (Battista et al., 2008), and the collector channels to occlude, leading to increased outflow resistance and primary open-angle glaucoma (POAG). To date, OCT is the only device that supports noninvasive and contrast-free imaging of SC with high resolution. OCT visualization has enabled precise measurements of SC, with its meridional diameter ranging from 127 to 283 μm and cross-sectional area ranging from 4,064 to 13,991 μm^2^ in the normal eyes (Shi et al., 2012; Wang et al., 2012; Hong et al., 2013; Chen et al., 2018a; Chen et al., 2018b). Our results in the normal control group are consistent with these findings. Using OCT, Kagemann et al. (2014b) found that acute IOP elevation significantly reduced the SC area in healthy eyes. Moreover, several previous studies (Wang et al., 2012; Hong et al., 2013; Yan et al., 2016; Yuan et al., 2021) showed that eyes with POAG had a lower SC area and smaller SC diameter than that in normal eyes. Our results align with those of *in vivo* human OCT studies. However, *in vivo* SC cross-sectional imaging varies considerably among different participants and regions of the same eye (Li P et al., 2017). The location of the scan line also has a potential impact on SC imaging. Thus, two single cross-sectional slices of SC may not represent the entire SC of the eye. Accordingly, the cross-sectional imaging protocol used in most previous studies might have a measurement bias in SC.

*En face* OCT, which is widely used in retinal and choroidal diseases (Gallo et al., 2022; Kongwattananon et al., 2022), has been used to visualize vasculatures. Recent advancements in hardware and software have facilitated the incorporation of posterior segment OCT technologies into anterior segment OCT systems, enabling imaging that is free from motion artifacts and providing enhanced resolution and depth penetration. Within the AS-OCT systems, the *en face* section, also known as the coronal section, is a reconstructed plane that is tangential to the limbus and perpendicular to the ocular surface. Unlike radial cross-sections, which provide a sectional view of the SC lumen, *en face* sections offer a circumferential representation of SC. Huang et al. (Huang et al., 2017) demonstrated the ability to perform a 360° three-dimensional reconstruction of SC in one healthy volunteer eye using a commercial SD-OCT instrument that included an active eye tracker to compensate for motion artifacts. Meanwhile, Yao et al. (Yao et al., 2021) utilized SS-OCT to perform complete circumferential imaging of SC in two healthy human eyes. They reconstructed three-dimensional renderings of SC by stitching the segmented SCs from the volumetric datasets. The entire imaging session and extensive post-acquisition processing required a duration of 1–2 days for completion. In our study, an ultrahigh-speed SS-OCT system with an eye-tracker technique and a commercial fully automated segmentation algorithm was used to acquire and rebuild motion-free *en face* images with a size of 6 mm × 6 mm in the nasal and temporal limbal area quadrants within a short period, which provides more clinical viability. On *en face* images of the deep layer, SC was detected as a dark curved line parallel to the corneal limbus. The location and structural morphology of SC are similar to those reported in previous histopathological studies (Moses, 1979; Hann et al., 2011). In the POAG group, the structure of SC in *en face* images was presented as a vague discontinuous curved line parallel to the corneal limbus, which is in agreement with previous studies. Allingham et al. (1996) examined five enucleated POAG eyes and showed that POAG eyes had more histological variability in SC, the canal could not be delineated, and the anterior portion of SC was more collapsed and closed, probably owing to trabecular meshwork herniation and collector channel obstruction.

Furthermore, the notion of the visible percentage of *en face* SC was introduced in our study because of the variations in SC. The percentage of visible SC in the entire pathway indicates the patency of SC lumen, which represents the outflow facilities of the aqueous humor. As expected, the visible percentage of SC in *en face* images in POAG eyes was significantly lower than that in normal eyes. The visible percentage of SC in the POAG group was reduced by 9.2% and 8.42% in the temporal and nasal quadrants, respectively, compared with that in the normal group. In addition, the relationships between IOP and the visible percentage or *en face* area of SC were investigated separately. The visible percentage and *en face* area of SC were negatively correlated with IOP as well as the cross-sectional area of SC in POAG patients. These results suggest that changes in the shape of SC in glaucomatous eyes may increase the resistance of the aqueous humor outflow leading to a vicious cycle of IOP elevation, which is consistent with previous research (Allingham et al., 1996; Wang et al., 2012). The finding of an inverse correlation between the parameters of SC in *en face* imaging and IOP is a novel observation that provides further evidence that the three-dimensional shape of SC is significantly associated with IOP and probably with even better sensitivity. Our findings further support the minimally invasive glaucoma surgeries that target SC to reduce IOP, such as dilation of SC or removal of the inner wall of SC based on the results of *en face* imaging to modify aqueous humor outflow.

Typically, visualizing SC in *en face* images has several advantages. First, the detection and localization of *en face* SC become more straightforward. Second, *en face* SC imaging provides a more holistic assessment of the aqueous outflow structure, especially in glaucomatous eyes. Third, this intuitive visualization of SC structure may guide clinicians to achieve the maximum reduction of IOP at the surgical site. However, our study also has the following limitations: First, we did not assess the effect of antiglaucomatous drugs on SC measurements. Most patients (39/50) received prostaglandin analog eye drops to lower the IOP, which might have affected SC assessments. Second, we obtained SC morphology and parameters only for the nasal and temporal quadrants because the superior and inferior sections had low visibility and poor imaging quality owing to the eyelid interference, so we did not apply this method in our research. Future work will include a more comprehensive circumferential morphological analysis of SC. Third, the sample size was relatively small, rendering it challenging to investigate SC morphology at different POAG stages. Future research will enroll more patients.

In conclusion, we demonstrated that 1,060 nm SS-OCT could be used to perform nasal and temporal *en face* SC imaging and quantification in both POAG and normal eyes. By avoiding extensive post-acquisition processing, *en face* SC imaging enabled a more sophisticated analysis of SC morphology than cross-sectional imaging alone. The *en face* imaging offers a new way of visualizing and evaluating SC in POAG. This technique may guide the glaucoma procedures targeting SC and help to assess the post-surgical improvement in SC in the future.

## Data Availability

The original contributions presented in the study are included in the article/Supplementary Material, further inquiries can be directed to the corresponding author.
